# Supplementary material for: Navigating challenges in radiography research: radiographers’ perspectives in Saudi Arabia
Source: PeerJ. 2024 Sep 20;12:e18125. doi: 10.7717/peerj.18125 (PMC11418811; doi:10.7717/peerj.18125)
Supplement: Supplemental Information 3 [file peerj-12-18125-s003.docx]

| Please click the link below to access the questionnaire: <https://forms.office.com/Pages/ResponsePage.aspx?id=LdCoE_NZakGCMbMIDmOcrWQIs4Obz0VCg6t6MP7poo5UQ01aMVNJSDJPQlhKWldRU0ZYU0lRQUNKUS4u> | |
| --- | --- |
| Q1. What is your gender? | 1. Male 2. Female |
| Q2. What is your age? | 1. 18-24 years 2. 25-34 years 3. 35-44 years 4. 45-54 years 5. 55-60 years |
| Q3. What is your discipline? | 1. Radiography 2. Others – please specify: |
| Q4. In which province do you currently work? | Eastern Region  Western Region  Middle Region  Northern Region  Southern Region |
| Q5. How would you best describe your current workplace? | Public hospital  Private hospital  Academic  Semi-public (King Faisal Specialist Hospital & Research Centre, National Guard Health Affairs, Military)  Others – please specify: |
| Q6. What is your current employment status? | Full time  On educational leave  Unemployed  Retired   1. Other (please specify) |
| Q7. How many years’ experiences do you have in the medical radiations profession? | 1. 0-5 years 2. 6-10 years 3. 11-15 years 4. 16-20 years 5. >21 years |
| Q8. What is your highest qualification related to medical radiations? | 1. Doctoral degree 2. Masters degree 3. Bachelor degree 4. Graduate diploma |
| Q9. What is Your main position | 1. Radiographer in clinical practice 2. Manager or equivalent 3. Other, what? (eg. PACS administrator) |
| Q10. Are you familiar with the research strategy at your workplace? | 1. Yes 2. No 3. I don’t know, if we have a research strategy 4. We don’t have a research strategy |
| Q11. Are you currently undertaking further study? | 1. Yes – go to question 12 2. No – go to question 14 |
| Q12. If so, what study are you undertaking? | 1. Doctoral degree 2. Masters degree 3. Bachelor degree |
| Q13. Is this study related to medical radiations? | 1. Yes 2. No – what field is this further study in? |
| Q14. Are you currently (or have you ) involved in any medical radiations related research? | 1. Yes – go to question 15 2. No – go to question 18 |
| Q15. If yes, which of the following research activities have you participated in at your workplace after graduating as a radiographer? (please select all that apply)? | 1. Planning the study 2. Writing the research protocol 3. Applying for project funding 4. Applying to ethical committee 5. Applying for hospital or other approval 6. Reviewing the background literature 7. Recruitment of participants 8. Collecting the data 9. Analyzing the data 10. Writing a scientific article 11. Presenting results in a conference 12. Other activity, identify please? |
| Q16. If yes, please indicate up to three most important reason(s) for undertaking the research activities at your workplace after graduating as a radiographer | 1. As a part of working task 2. For professional development 3. To enhance promotion prospects 4. For economic interest 5. To improve patient care 6. To provide evidence based radiography practice 7. To advance the profession of radiographers 8. Other reason, identify please? |
| Q17. If Yes, approximately in how many research projects have you been involved? | 1. under 5 2. 5-10 3. more than 10 |
| Q18. If No, please indicate up to three reason(s) NOT undertaking the research activities at your workplace after graduating as a radiographer | 1. Research is not a part of my working task 2. Lack of time to engage in research at my workplace 3. There is no research culture at my workplace 4. I am not aware of the potential research projects to participate. 5. I don’t have ideas for research projects 6. I don’t have sufficient skills for participating in research projects 7. I am not interested in participating in research projects 8. I don’t see any benefit in participating in research projects 9. Other reason, identify please? |
|  |  |
| Q19. For each statement, please choose the response that best describes your opinion (5-points scale) | 1. I have sufficient knowledge about scientific research process 2. I have sufficient skills to search background literature 3. I have sufficient skills to critically evaluate research articles 4. I have sufficient English language skills 5. I have sufficient knowledge about research methodology 6. I have sufficient knowledge about statistical analyses 7. I am capable of participating in radiography research project 8. I am capable of initiating a radiography research project |
| Q20. Which are the three most important factors of the following that promote or might promote your participation in research projects? | 1. I get research training opportunities 2. I get assigned working time to conduct research 3. I get funding and other material resources 4. I receive support from my colleagues (other radiographers) 5. I receive support from department manager and/or higher management 6. I receive support from other professionals (academics, physicists, radiologists) 7. Employing experienced radiography researchers for mentorship 8. Recognition at organizational level 9. Being a member of a research group 10. Other factor, identify please? |
| Q21. Which are the three most essential factors of the following that prevent or might prevent your participation in research projects? | 1. Lack of knowledge and skills to conduct research 2. Insufficient time at work to conduct research 3. Lack of interest and motivation 4. Lack of funding and other material resources 5. Lack of support from my colleagues (other radiographers) 6. Lack of support from department manager and/or higher management 7. Lack of support from other professionals (academics, physicists, radiologists) 8. Lack of knowledge about potential research projects to participate 9. Lack of experienced radiography research mentors 10. Lack of radiography research culture at workplace 11. Other factors, identify please? |
| Q22. For each statement, please choose the response that best describes your opinion (5-points scale) | 1. Research is needed in radiography to promote the radiography profession 2. Research is needed in radiography to provide the evidence base for radiographic practice 3. Clinical decisions in radiographic practice should be based on research evidence 4. Radiographers with a radiography education are competent to conduct radiography research 5. Radiographers with a master’s degree are competent to conduct radiography research 6. Radiographers with a doctoral/licentiate degree are competent to conduct radiography research 7. Radiographers should be initiators of radiographic research projects 8. Radiographers should be in charge of radiographic research projects 9. Radiographic research projects should be initiated and led by healthcare institutions only 10. Radiographic research projects should be initiated and led by educational institutions only 11. Cooperation between educational and healthcare institutions in conducting radiography research is important |
| Q23. Please provide details of the number of items in each category you have been involved in over the last 5 years | 1. Published journal articles 2. Grant applications (successful and unsuccessful) 3. Research student supervision 4. Presentations (oral and poster) 5. Published book chapters 6. Other (please provide details) 7. None of the above |
| Q24. Do you have any further comments on research in the medical radiations profession, barriers and/or further support mechanisms that could be provided to promote research activity in the profession? |  |
