# Supplementary material for: Navigating challenges in radiography research: radiographers’ perspectives in Saudi Arabia
Source: PeerJ. 2024 Sep 20;12:e18125. doi: 10.7717/peerj.18125 (PMC11418811; doi:10.7717/peerj.18125)
Supplement: Supplemental Information 4 [file peerj-12-18125-s004.pdf]

استبيان لاستطلاع آراء أخصائيي/فنيي الأشعة في المملكة العربية السعودية بشأن الأبحاث العلمية

PV1.0

السلام عليكم ورحمة الله وبركاته

ندعوكم لتشاركونا جزءًا كبيرًا وجوهريًا من بحث بعنوان:

اراء أخصائي / فنيي الأشعة في المملكة العربية السعودية بشأن الأبحاث العلمية

وذلك عن طريق إجابتكم لهذا الاستبيان.

تهدف الدراسة لمعرفة آراء ووجهات نظر أخصائي وفنيي الأشعة التشخيصية بالأبحاث العلمية، مع التركيز على معرفة الصعوبات التي قد تواجههم في المشاركة في الأبحاث العلمية، سعيا لتطوير وتنمية المعرفة والسلوكيات الإيجابية تجاه الأبحاث العلمية

قد يستغرق استكمال الاستبيان 5-10 دقائق علما بأن الاستبيان تفاعلي وقد تختلف الأسئلة بناء على إجابات المشارك

للاستفسار بخصوص البحث او الاستبيان، يمكنكم التواصل مع مشرف البحث (د. فيصل الرحيلي) عن طريق البريد الالكتروني التالي

frehily@taibahu.edu.sa

شاكرين لكم حُسن تعاونكم.

* Required

1. هل توافق على المشاركة في الدراسة؟ *

- ◯ نعم
- ◯ لا

2. الجنس *

- ◯ ذكر
- ◯ أنثى

3. العمر (سنة) *

- 18-24
- 25-34
- 35-44
- 45-54
- 55-60

4. التخصص *

- التصوير الطبي (الأشعة التشخيصية)
- آخر ، الرجاء ذكره في الخانة السفلى
- Other

5. في أي منطقة تعمل حاليا؟ *

- المنطقة الشرقية
- المنطقة الغربية
- المنطقة الوسطى
- المنطقة الشمالية
- المنطقة الجنوبية

6. نوع مكان العمل *

- مستشفى حكومي
- مستشفى شبه حكومي (مستشفى الملك فيصل التخصصي ومركز الأبحاث، الشؤون الصحية بالحرس الوطني، المستشفيات العسكرية)
- مستشفى خاص
- أكاديمي
- آخر ، الرجاء ذكره في الخانة السفلى
- Other

7. ما هي الحالة الوظيفية حالياً؟ *

- ◯ دوام كامل
- ◯ اجازة تعليمية (ايفاد/ ابتعاث)
- ◯ عاطل عن العمل
- ◯ متقاعد

8. كم عدد سنوات خبرتك في مهنة التصوير الطبي (الأشعة التشخيصية)؟ *

- ◯ ٠-٥ سنوات
- ◯ ٦-١٠ سنوات
- ◯ ١١-١٥ سنة
- ◯ ١٦-٢٠ سنة
- ◯ ٢١ سنة<

9. ما هي أعلى مؤهلاتك المتعلقة بالتصوير الطبي (الأشعة التشخيصية)؟ *

- ◯ درجة الدكتوراه
- ◯ درجة الماجستير
- ◯ درجة البكالوريوس
- ◯ دبلوم

10. ما هو منصبك الرئيسي؟ *

- ◯ أخصائي/فني التصوير الطبي (الأشعة التشخيصية)
- ◯ مدير او ما يعادله
- ◯ اخرى، يرجي ذكرها في الخانة السفلى (مثلا مسؤول عن نظام الباكس)
- ◯ Other

11. هل أنت على دراية باستراتيجية البحث العلمي في مقر عملك؟ *

- ◯ نعم
- ◯ لا
- ◯ لا اعلم اذا كان يوجد لدينا استراتيجية بحث

12. هل تدرس حالياً؟ *

- ◯ نعم
- ◯ لا

13. ما هي نوع الدراسة التي تقوم بها؟ *

- ◯ درجه الدكتوراه
- ◯ درجه الماجستير
- ◯ درجه البكالوريوس

14. هل هذه الدراسة مرتبطة بالتصوير الطبي (الأشعة التشخيصية)؟ *

- ◯ نعم
- ◯ لا، اذكر المجال في الخانة السفلى
- ◯ Other

15. هل أنت حاليا مشارك (أو سبق وأن شاركت) في أي أبحاث متعلقة بالتصوير الطبي (الأشعة التشخيصية)؟ *

- ◯ نعم
- ◯ لا

16. أي من الأنشطة البحثية التالية سبق لك المشاركة فيها في مكان عملك بعد التخرج كأخصائي أشعة؟ (يمكن اختيار عدة اجابات) *

- ☐ التخطيط للبحث او الدراسة - Planning the study
- ☐ كتابة بروتوكول البحث - Writing the research protocol
- ☐ التقدم بطلب تمويل المشروع البحثي - Applying for project funding
- ☐ التقديم على اللجنة الأخلاقية - Applying for ethical committee
- ☐ التقدم بطلب للحصول على موافقة المستشفى أو غيرها - Applying for hospital or other approval
- ☐ مراجعة الاداب و الدراسات المرجعية - Reviewing the background literature
- ☐ توظيف المشاركين - Recruitment of participants
- ☐ جمع البيانات - Collecting the data
- ☐ تحليل البيانات - Analyzing the data
- ☐ كتابة مقال علمي - Writing a scientific article
- ☐ عرض النتائج في مؤتمر - Presenting results in a conference
- ☐ نشاط آخر، اذكره في الخانة السفلى
- ☐ Other

17. رجاءآ اختر ثلاثة أهم أسباب دفعتك للقيام بالأنشطة البحثية في مكان عملك بعد التخرج كأخصائي أشعة؟ *

Please select at most 3 options.

- ☐ جزء من مهام العمل - As a part of working task
- ☐ للتطور و التقدم المهني الاحترافي - For professional development
- ☐ لتعزيز آفاق الترقية - To enhance promotion prospects
- ☐ لمصلحة اقتصادية - For economic interest
- ☐ لتحسين رعاية المرضى - To improve patient care
- ☐ توفير ممارسه لتصوير الطبي (الأشعة التشخيصية) القائمة على الأدله - To provide evidence based radiography practice
- ☐ تطوير و تحسين مهنة لتصوير الطبي (الأشعة التشخيصية) - To advance the profession of radiographers
- ☐ سبب آخر، يرجى ذكره في الخانة السفلى؟
- ☐ Other

18. كم عدد المشاريع البحثية التي سبق أن شاركت بها؟ *

- اقل من ٥
- ٥ الى ١٠
- اكثر من ١٠

19. رجاءاً، اختر ثلاث اسباب لعدم مشاركتك او اتخاذك لدور بحثي (يمكن اختيار عدة اجابات *

Please select at most 3 options.

- المشاركة في الأبحاث ليست من مهامي الوظيفية
- عدم توفر الوقت للمشاركة في الأبحاث
- عدم وجود ثقافة البحث في مكان العمل
- لست على علم بالمشاريع البحثية المحتملة للمشاركة بها
- لست املك أفكار لمشاريع بحثية
- لست املك المهارة الكافية للمشاركة في المشاريع البحثية
- لا أهتم بالمشاركة في المشاريع البحثية
- لست أرى أي فائدة في المشاركة في المشاريع البحثية
- أسباب اخرى, يرجى ذكرها في الخانة السفلى
- Other

20. يرجى تحديد مدى موافقتك على العبارات التالية باستخدام المقياس التالي

موافق بشدة (5)
موافق (4)
محايد (3)
غير موافق (2)
غير موافق بشدة (1)

يرجى ملاحظة أن "**5**" يعني أنك تتفق تمامًا مع العبارة، وأن "**1**" يعني أنك لا تتفق تمامًا مع العبارة *

	5	4	3	2	1
املك معرفة كافية حول الأبحاث العلمية و طريقة سيرها	◯	◯	◯	◯	◯
املك مهارة كافية للبحث في الدراسات\ الآداب المرجعية (المسح الأدبي)	◯	◯	◯	◯	◯
املك المهارة النقدية الكافية لتقييم المقالات البحثية	◯	◯	◯	◯	◯
املك مهارة لغوية كافية في اللغة الإنجليزية	◯	◯	◯	◯	◯
املك معرفة كافية حول الطرق البحثية	◯	◯	◯	◯	◯
املك معرفة كافية حول التحليل الإحصائي	◯	◯	◯	◯	◯
انا متمكن من المشاركة في مشاريع بحثية متعلقة بالتصوير الطبي (الأشعة التشخيصية)	◯	◯	◯	◯	◯
انا متمكن من المبادرة ببدأ مشروع بحثي متعلق بالتصوير الطبي (الأشعة التشخيصية)	◯	◯	◯	◯	◯

21. ماهي أهم العوامل التي تحفزك او قد تحفزك للمشاركة في المشاريع البحثية؟ (يمكن اختيار عدة اجابات) *

- [ ] أحصل على فرص التدريب البحثي
- [ ] أحصل على وقت عمل مخصص للقيام بالادوار البحثية
- [ ] أحصل على تمويل و موارد مادية اخرى
- [ ] اتلقى الدعم من زملائي في العمل (أخصائي/فني التصوير الطبي (الأشعة التشخيصية))
- [ ] اتلقى الدعم من مدير القسم او الادارة العليا
- [ ] اتلقى الدعم من مهنيين اخرون (الفيزيائيون او اطباء الاشعة او الاكاديميون)
- [ ] توظيف ذوي الخبرة من الباحثين في مجال التصوير الطبي (الأشعة التشخيصية) لأدوار ارشادية
- [ ] الحصول على التقدير من المنظمة
- [ ] أن أكون عضوا في مجموعة بحثية

22. ماهي اهم الأسباب التي تمنعك او قد تمنعك من المشاركة في المشاريع البحثية؟ (يمكن اختيار عدة اجابات) *

- [ ] قلة المعرفة و المهارات اللازمة
- [ ] قلة الوقت في العمل
- [ ] قلة الإهتمام و الحافز
- [ ] قلة التمويل و الموارد المادية الاخرى
- [ ] قلة الدعم من الزملاء في العمل(أخصائي/فني التصوير الطبي (الأشعة التشخيصية))
- [ ] قلة الدعم من مدير القسم او الادارة العليا
- [ ] قلة الدعم من مهنيين اخرون (الفيزيائيون او اطباء الاشعة او الاكاديميون)
- [ ] قلة المعرفة حول المشاريع البحثية المحتملة التي يمكن المشاركة بها
- [ ] قلة المرشدين/ المشرفين ذوي الخبرة
- [ ] قلة او انعدام ثقافة البحث في مكان العمل
- [ ] اسباب اخرى، يرجي ذكرها في الخانة السفلى
- [ ] Other

23. يرجى تحديد مدى موافقتك على العبارات التالية باستخدام المقياس التالي

موافق بشدة (5)
موافق (4)
محايد (3)
غير موافق (2)
غير موافق بشدة (1)

يرجى ملاحظة أن "**5**" يعني أنك تتفق تمامًا مع العبارة، وأن "**1**" يعني أنك لا تتفق تمامًا مع العبارة *

	5	4	3	2	1
اجراء الابحاث مطلوب لتنمية و تعزيز مهنة التصوير الطبي (الأشعة التشخيصية)	○	○	○	○	○
اجراء الأبحاث مطلوب لتوفير الممارسة القائمة على الأدلة في مجال التصوير الطبي (الأشعة التشخيصية)	○	○	○	○	○
القرارات السريرية عند ممارسة التصوير الطبي (الأشعة التشخيصية) يجب ان تكون مبنية على الأدلة البحثية	○	○	○	○	○
أخصائيي/فنيي التصوير الطبي (الأشعة التشخيصية) الحاصلون على درجة تعليمية في التصوير الطبي مؤهلون لاجراء الابحاث في المجال	○	○	○	○	○
أخصائيي/فنيي التصوير الطبي (الأشعة التشخيصية) الحاصلون على درجة الماجستير في التصوير الطبي مؤهلون لاجراء الابحاث في المجال	○	○	○	○	○
أخصائيي/فنيي التصوير الطبي (الأشعة التشخيصية) الحاصلون على درجة الدكتوراة في التصوير الطبي مؤهلون لاجراء الابحاث في المجال	○	○	○	○	○
يجب ان يكونوا أخصائيي/فنيي التصوير الطبي (الأشعة التشخيصية) هم المبادرين للمشاريع البحثية في المجال	○	○	○	○	○
يجب ان يكونوا أخصائيي/فنيي التصوير الطبي (الأشعة التشخيصية) هم المسؤولين في [illegible]	○	○	○	○	○

المشاريع البحثية في المجال

24. رجاءً، اختر أنواع المشاركات التي قمت بها خلال الخمس سنوات الماضية (يمكن ا *

يجب ان تكون مؤسسات الرعاية الصحية وحدها فقط هي المسؤولة عن بدأ وقيادة المشاريع البحثية في مجال التصوير الطبي (الأشعة التشخيصية)

يجب ان تكون المؤسسات التعليمية وحدها فقط هي المسؤولة عن بدأ وقيادة المشاريع البحثية في مجال التصوير الطبي (الأشعة التشخيصية)

التعاون بين المؤسسات التعليمية ومؤسسات الرعاية الصحية في اجراء الأبحاث في مجال التصوير الطبي (الأشعة التشخيصية) يعد امرا مهما

- نشر
- طلبات المنح (الناجحة
- اشراف على ال
- العروض التقديمية (الشفو
- فصل في
- لا
- اخرى، يرجى ذكرها في ا

25. هل لديك أي تعليقات أخرى بشأن الابحاث في مجال التصوير الاشعاعي كالعوائق أو [illegible]
[illegible] يمكن أن تعزز النشاط البحثي في [illegible]

This content is neither created nor endorsed by Microsoft. The data you submit will be sent to the form owner.

Microsoft Forms
